# Mathematical Logic in the Human Brain: Semantics

**DOI:** 10.1371/journal.pone.0053699

**Published:** 2013-01-03

**Authors:** Roland M. Friedrich, Angela D. Friederici

**Affiliations:** 1 Max Planck Institute for Human Cognitive and Brain Sciences, Leipzig, Germany; 2 Institute for Mathematics, Humboldt University Berlin, Berlin, Germany; National Research & Technology Council, Argentina

## Abstract

As a higher cognitive function in humans, mathematics is supported by parietal and prefrontal brain regions. Here, we give an integrative account of the role of the different brain systems in processing the semantics of mathematical logic from the perspective of macroscopic polysynaptic networks. By comparing algebraic and arithmetic expressions of identical underlying structure, we show how the different subparts of a fronto-parietal network are modulated by the semantic domain, over which the mathematical formulae are interpreted. Within this network, the prefrontal cortex represents a system that hosts three major components, namely, control, arithmetic-logic, and short-term memory. This prefrontal system operates on data fed to it by two other systems: a premotor-parietal top-down system that updates and transforms (external) data into an internal format, and a hippocampal bottom-up system that either detects novel information or serves as an access device to memory for previously acquired knowledge.

## Introduction

Processing mathematical formulae is a cognitive task that most of us are required to perform every day. The more abstract the formulae, the more difficult their processing seems to be. To date, it has never been clarified why more abstract realizations of a common underlying structure are perceived as being more difficult to process than more concrete ones, even though the formulae are structurally isomorphic. If it is not the structure, the difference must lie in the semantic domain over which the formulae are interpreted. This hypothesis is tested in the present study.

Previous research has already investigated the neural basis of number processing quite extensively: either as single items or in simple arithmetic and algebraic calculations [1], [2], [3], [4]. The intraparietal sulcus (IPS) was found to systematically activate for all these number-related tasks. Therefore, this region was taken as a key region for the representation of numerical quantity (for recent reviews see [5] or [6]). Neuroimaging research on the processing of syntax in mathematics is sparse. A recent study on the processing of different structural hierarchies in mathematical formulae found that the ventral portion of the left anterior inferior frontal gyrus (Brodmann Area [BA] 45/47), in addition to the bilateral middle temporal and inferior parietal regions, showed increased activation in response to complex hierarchical compared to flat structures [7]. Activation of some frontal regions, i.e., the medial frontal gyrus, the middle frontal gyrus (BA 6), and also BA 47, varied as a function of the formulae's incorrectness and must thus be viewed not to be specifically tied to the processing of structural hierarchy, but rather to process structured formulae in general. These brain regions in the prefrontal cortex, although crucial for processing mathematical formulae, are not specific to the domain of mathematics. Rather, the prefrontal cortex has been allocated to aspects of cognitive control [8], [9], [10], [11] and working memory [12], [13], [14]. These two aspects, cognitive control and working memory, are also relevant for the processing of structured sequences [15], [16], [17], and for the judgment of relations in the language and non-language domains [18], [19], respectively.

Here, we focused on the neural basis of processing the semantic content of mathematical formulae, i.e., their truth value (Boolean value). Therefore, we designed a functional magnetic resonance imaging (fMRI) experiment, using syntactically well-formed hierarchical formulae written in a standard first-order language. The formulae themselves were interpreted in either the domain of abstract algebra or arithmetic, i.e., without numbers or with integer numbers. Participants were required to read these formulae and to decide whether they were true or false statements. In contrast to our previous study on syntax, where we used formulae in which the first occurrence of a grammatical error rendered the whole expression incorrect, and no further processing was required to achieve a well-formed judgment, in this study the calculation of the Boolean truth value needed the evaluation of the entire phrase.

By keeping the structures identical but varying the domain or the truth value, we expected to disentangle the specific roles of various prefrontal areas with respect to cognitive control and working memory and, further, to shed light on the neural substrate of processing difficulty. We expected to find a bilateral fronto-parietal network when comparing the different algebraic and arithmetic conditions, consisting of regions previously identified to be involved in the processing of the syntax of hierarchical expressions, and domain specificity manifesting itself as local differences in activation strength or volume extension. Moreover, we predicted a differential time course of activations for brain regions involved in calculating the truth value of the formulae depending on the semantic domain. Therefore, two separate analyses were conducted: one for activations 4 s post stimulus and another for activations 8 s post stimulus presentation.

## Results

### Behavioral results

The percentage of correct answers given was very high. For the four different formulae conditions, 14 out of 15 participants achieved the following scores: algebra true, mean  = 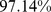, SD  = 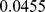; algebra false, mean  = 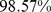, SD  = 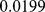; arithmetic true, mean  = 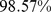, SD  = 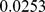; arithmetic false, mean  = 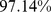, SD  = 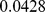. The pair-wise differences for the percentage of correct answers given across the four stimuli sub-types were not significant at 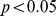 (calculated using a paired 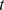-test).

### fMRI results

In all tables and figures, coordinates 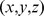, are reported in three-dimensional Talairach space [20]. Capital 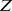 refers to the statistical 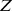-value, which is also used to give the numerical values of local activation extrema. Finally, volumes of activation clusters are denoted in cubic millimeters (mm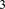).

### Active base network

To isolate the entire network of regions involved in the active tasks, we compared the blood-oxygen-level-dependent (BOLD) activity for each of the four formulae conditions and the baseline trials (no task).

**Figure 1:**
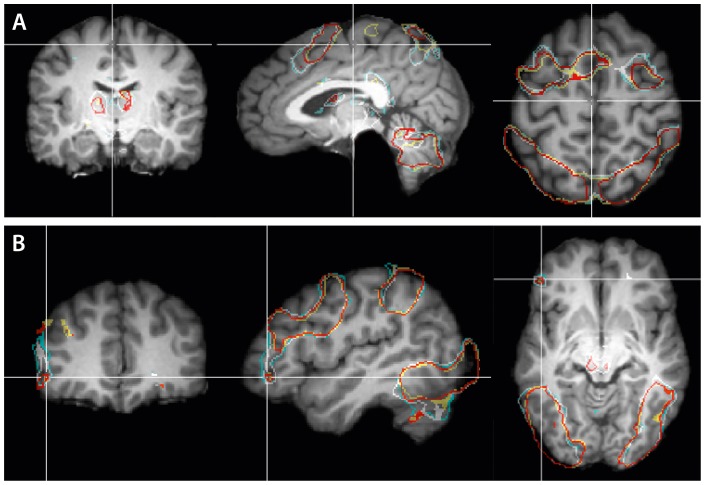
Boundaries of activation clusters. The figure shows the boundaries of activation clusters separately for each mathematical condition compared to baseline, corresponding to 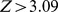 (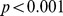 uncorrected) and mapped onto a reference brain (single subject). The colors represent: true algebraic (light blue), false algebraic (white), true arithmetic (yellow) and false arithmetic (red) conditions. (A) Top row from left to right: coronal (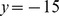), sagittal (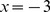) and axial (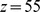) section. (B) Bottom row from left to right: coronal (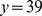), sagittal (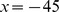) and axial (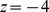) section.

Significant brain activation was observed for all formulae compared to the baseline, in an extended fronto-parietal network, which included the parietal, dorsal occipital, inferior temporal, premotor and prefrontal cortices, as well as the insula bilaterally. Further, we found subcortical structures such as the basal ganglia and the thalamus to be involved (see Figure 1). These subcortical structures are known to be functionally and structurally linked to the prefrontal cortices [21], [22], [23], [24].

Having defined the base network, we then probed how it was specifically activated with respect to the domain (algebra vs. arithmetic), the sub-types (true vs. false), and the temporal evolution (early period, i.e., 4 s after stimulus onset vs. long period, i.e. 8 s after stimulus onset).

### Algebra true vs. arithmetic true

To allow a direct comparison between semantic domains which is independent of possible different truth values, we compared the true algebraic and the true arithmetic conditions. We assumed that the BOLD signals in the early period would mainly reflect transient encoding activations, whereas the long period would be dominated by control, arithmetic-logical and maintenance operations.

**Figure 2:**
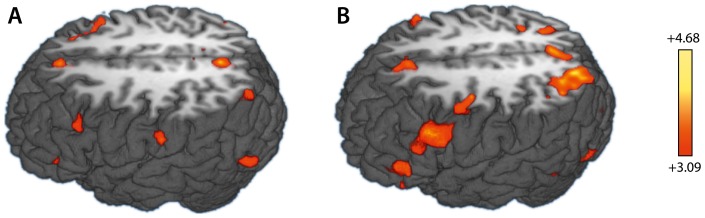
Functional activations for algebra vs. arithmetic. A: top left-lateral view of the functional activations 4 s after stimulus onset for the contrast “algebra true vs. arithmetic true”, overlaid onto a three-dimensional rendering of the brain of a representative individual, axially cut at 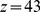. B: top left-lateral view of the functional activations 8s after stimulus onset for the contrast “algebra true vs. arithmetic true”, overlaid onto a three-dimensional rendering of the same brain as in A, axially cut at 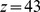. The color bar indicates 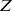-values (uncorrected) and applies to both A and B.

For the early period, i.e., the first 4 s after stimulus onset, the comparison of BOLD signals of algebraic and arithmetic conditions revealed a number of regions to show increased activation for algebra. In the prefrontal cortex there are two foci in the lateral aspects of the left and right middle frontal gyri, (BA 8/6). In the parietal cortex, an extended region was activated, which involved the ventral parts of the precuneus (PCU) and the adjacent portion of the dorsal posterior cingulate area (BA 31), but did not extend into the superior parietal lobule (SPL). In addition, the right middle temporal gyrus (BA 39), the left fusiform gyrus (BA 37), and the bilateral parahippocampal gyri (PhG) were more active for algebra than for arithmetic. Further, we found the basal ganglia (including the caudate nucleus and the globus pallidus) and the thalamus to be significantly more involved (see Table 1, Figure 2A).

**Table 1:** Algebra true vs. arithmetic true (early period: 4 s after stimulus onset).

AREA	Talairach coordinates								
	left			right			*z*_max_	*z*_mean_	mm^3^
MFG, BA 8/6	−29	18	48	–	–	–	**4.77**	3.47	648
MFG, BA 8/6	–	–	–	28	16	45	**3.82**	3.38	567
Pcu, BA 7	–	–	–	4	−60	30	**4.19**	3.48	3321
MTG, BA 39	–	–	–	40	−60	27	**4.19**	3.53	594
FuG, BA 37	−35	−42	−12	–	–	–	**5.15**	3.62	2214
PhG, BA 27	−23	−30	−6	–	–	–	**3.73**	3.36	297
PhG, BA 27	–	–	–	22	−30	−6	**3.73**	3.30	486

Activations for the contrast: “algebra true vs. arithmetic true”, thresholded at 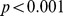 (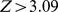 uncorrected) and with an extent of at least 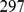
mm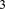. Contrast is evaluated for the BOLD signal for 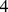 s after stimulus onset. BA  =  Brodmann area, FuG  =  fusiform gyrus, MFG  =  middle frontal gyrus, MTG  =  middle temporal gyrus, PhG  =  parahippocampal gyrus, Pcu  =  precuneus, 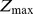: maximal 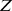-score, 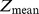: average 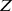-value at cluster level, volume in mm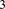.

For the long period, i.e., an interval of 8 s after stimulus onset, the same contrast of the BOLD signals revealed significant activation differences with more activation for algebra than for arithmetic in the frontal and parietal regions of the cortex. In the frontal cortex, this included the left inferior frontal gyrus (IFG; BA 47), the left (BA 9), the bilateral dorsolateral prefrontal cortex (DLPFC; BA 46/9), the bilateral premotor cortex (BA 6), and dorsocaudal portions of the medial frontal cortex (dACC; BA 32/8). In the posterior part of the brain, we found the ventral left and right inferior temporal gyrus (ITG; BA 37), the dorsal left and right inferior parietal lobuli, the precuneus, and the right superior parietal lobule (SPL; BA 7) to be more activated for algebra than for arithmetic (see Table 2 and Figure 2B).

**Table 2:** Algebra true vs. arithmetic true (long period: 8 s after stimulus onset).

AREA	Talairach coordinates								
	left			right			*z*_max_	*z*_mean_	mm^3^
IFG, BA 47	−43	33	0	–	–	–	**4.16**	3.68	1053
MFG, BA 46	−47	30	18	–	–	–	**3.90**	3.43	351
DLPC, BA 9	−50	21	33	–	–	–	**4.61**	3.56	1944
DLPC, BA 46/9	–	–	–	43	22	27	**4.28**	3.49	1836
mFG, BA 32,	−8	36	42	–	–	–	**4.87**	3.64	972
MFG, BA 6	−32	9	54	–	–	–	**4.50**	3.43	3429
MFG, BA 6	–	–	–	31	3	54	**3.93**	3.46	864
SFG, BA 6	−11	12	63	–	–	–	**4.08**	3.47	324
SPL, BA 7	–	–	–	25	−57	57	**4.03**	3.35	432
Pcu, BA 7	−23	−66	39	–	–	–	**4.09**	3.40	3294
mPcu, BA 7	−5	−63	39	–	–	–	**4.50**	3.50	2349
Pcu, BA 7	–	–	–	22	−69	36	**4.12**	3.36	810
FuG, BA 37	−38	−57	−9	–	–	–	**4.10**	3.53	486
MT+	−41	−72	−6	–	–	–	**3.94**	3.39	513

Activation maxima for the contrast: “algebra true vs. arithmetic true”, thresholded at 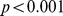 (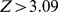 uncorrected) and with an extent of at least 297 mm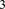. Contrast is evaluated for the BOLD signal 8 s after stimulus onset. BA  =  Brodmann area, DLPFC  =  dorsolateral prefrontal cortex, FuG  =  fusiform gyrus, IFG  =  inferior frontal gyrus, mFG  =  medial frontal gyrus, MFG  =  middle frontal gyrus, mPcu  =  medial precuneus, MT+  =  middle temporal complex, Pcu  =  precuneus, SFG  =  superior frontal gyrus, SPL  =  superior parietal lobule. 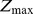: maximal 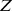-score, 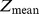: average 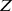-value at cluster level, volume in mm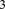.

### False vs. true formulae

The comparison of “all false vs. all true” formulae revealed that for the 4 s interval after stimulus onset, there were no significant activation differences (thresholded at 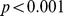, uncorrected, and with an extent of at least 297 mm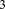). When analyzing the contrast “algebra false vs. algebra true”, with the BOLD signal up to 8 s after onset, we found no significant activation differences (thresholded at 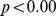, uncorrected, and with an extent of at least 297 mm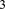). However, the contrast “arithmetic false vs. arithmetic true”, for the same time interval of 8 s after stimulus onset, yielded significant activation differences in parietal and frontal regions (see Table 3).

**Table 3:** Arithmetic false vs. arithmetic true (long period: 8 s after stimulus onset).

AREA	Talairach coordinates								
	left			right			*z*_max_	*z*_mean_	mm^3^
IFG, BA 47	−41	33	0	–	–	–	**4.00**	3.46	297
DLPC, BA 9	−55	12	30	–	–	–	**4.19**	3.54	2511
DLPC, BA 9	–	–	–	43	18	36	**3.94**	3.43	1026
MFG, BA 6	−44	0	54	–	–	–	**4.18**	3.45	432
IPL, BA 40	–	–	–	37	−36	45	**4.45**	3.59	1296
SPL, BA 7	−23	−69	45	–	–	–	**3.83**	3.42	324
SPL, BA 7	–	–	–	28	−63	51	**4.44**	3.47	1215
FuG, BA 37	–	–	–	31	−45	−9	**4.08**	3.43	432
MOG, BA 18	−23	−87	18	–	–	–	**4.41**	3.50	675

Activation maxima for the contrast: “arithmetic false vs. arithmetic true”, thresholded at 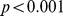 (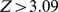 uncorrected) and with an extent of at least 297 mm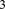. Contrast is evaluated for the BOLD signal 8 s after stimulus onset. BA  =  Brodmann area, DLPFC  =  dorsolateral prefrontal cortex, FuG  =  fusiform gyrus, IFG  =  inferior frontal gyrus, IPL  =  inferior parietal lobule, MOG  =  middle occipital gyrus, MFG  =  middle frontal gyrus, PcG  =  precentral gyrus, SPL  =  superior parietal lobule. 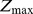: maximal 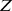-score, 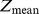: average 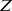-value at cluster level, volume in mm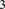.

The separate analyses for the two time windows suggested that the dorsolateral prefrontal cortex (BA 9), the medial and middle frontal cortex (BA 8, BA 46), and the inferior frontal gyrus (BA 47) only come into play during a later processing stage; a stage when the actual truth value is calculated or re-evaluated.

### Time course analysis for ROIs

**Figure 3:**
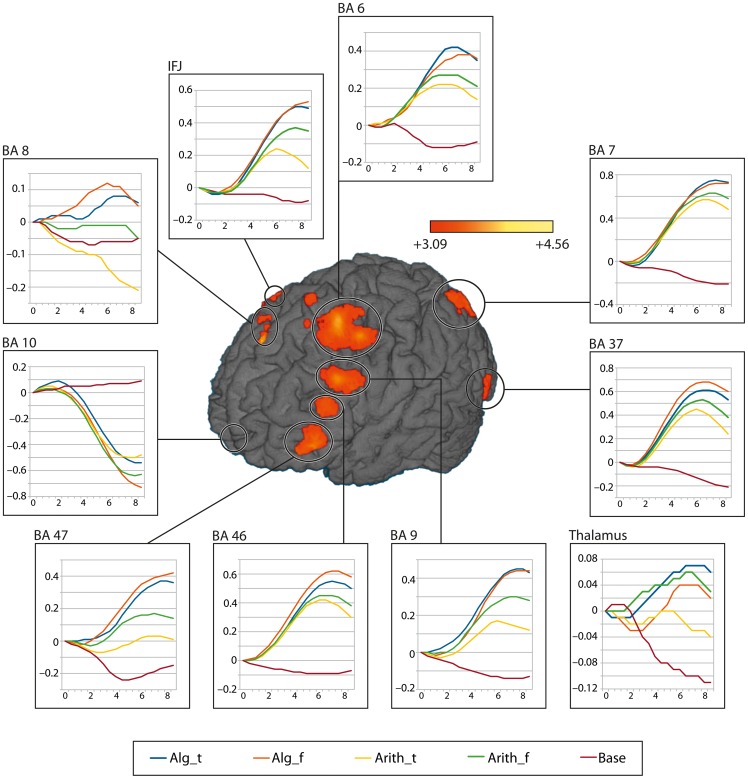
Time course of activation for different brain regions. Anterior-left lateral view of the functional activations for the contrast “algebra true vs. arithmetic true”, evaluated 8s after stimulus onset, and overlaid onto a three-dimensional rendering of the brain of a representative individual. The color bar indicates 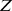-values (uncorrected). The curves represent percent signal changes associated with the true algebraic (blue), false algebraic (red), true arithmetic (yellow), false arithmetic (green) and baseline (wine red) conditions, averaged over a volume within each anatomical region and subjects. The x-axis shows time measured from stimulus onset in seconds and the 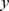-axis shows the normalized percent signal change.

In a next step we focused on the time course of activation in 10 regions of interest (ROI). The regions of interest were centered, in Talairach coordinates 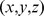 at: 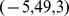, BA 10; 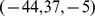, BA 47; 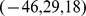, BA 46; 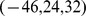, BA 9; 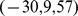, BA 6; 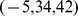, BA 8; 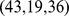 right inferior frontal junction region (IFJ); 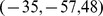, BA 7; 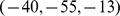, BA 37 (fusiform gyrus); 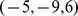, thalamus (see Figure 3). These regions had previously been discussed to be involved in processing formulae (BA 10, BA 6, BA 47, BA 7), reasoning (BA 47), and cognitive control (DLPFC), in reading (fusiform gyrus) and in mediating information (thalamus) in the literature [4], [7], [8], [25], [26], [27]. The average time course of the hemodynamic response for these ROIs for all 15 participants, and for all five conditions was evaluated. In such an analysis, differences in time to peak can be interpreted as differences in processing speed, whereas differences in amplitude indicate the amount of involvement of the particular region. Within each region, guided by the specifications we took from the literature, e.g., peak 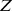-values, we determined a nearby Talairach coordinate, which served as the center of the cubic region, such that it was close to the nearest gray matter with respect to our anatomical data. All cubes measured 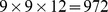 mm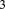 and were made out of the 26 adjacent voxels around each center.

The percentage change of the BOLD signal for every time series of each individual subject was evaluated for a period of 9 s after stimulus onset, and interpolated with an interval of 500 ms. Then the individual time series were averaged over all participants volume-wise. Subsequently, all time series were normalized, such that each one initiated at zero at the onset.

The time course of activation across the 8 seconds informs on the involvement of the 10 different brain regions for the true and false algebraic and arithmetic conditions and the baseline. While some of the regions do not show differences between the conditions neither in the early nor in the late period (BA 10) others indicate a shift of involvement from the early to the late period. These shifts are discussed in more detail below.

## Discussion

This fMRI study investigated differences in the processing of the truth value of structurally identical and syntactically well-formed hierarchical mathematical formulae of two different types: abstract algebra and integer numbers. Therefore, the resulting activation differences in processing these formulae should be domain-specific and not structure-specific. Further, we focused on determining the base network (active tasks vs. low-level baseline) involved and the relative dissimilarities (algebra vs. arithmetic), particularly with respect to the temporal evolution (early vs. extended period) of processing.

### Major cortical networks: attention, working memory and cognitive control

The human brain is organized into different and partially competing cortical networks [4], [8], [25], [28], [29]. This intrinsic organization has to be taken into account in order to interpret the fMRI activations recorded in the present study.

**Figure 4:**
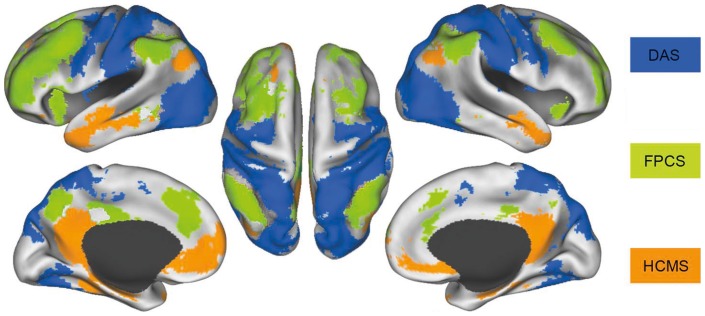
Intrinsically defined brain system. Intrinsically defined dorsal attention (DAS), frontoparietal control (FPCS), and hippocampal-cortical memory (HCMS) systems. Voxels in the DAS include regions correlated with MT+ and SPL and are shown in blue. Voxels in the FPCS include regions correlated with aPFC and aIPL and are shown in light green. Voxels in the HCMS include regions correlated with HF and pIPL and are shown in orange. Data are displayed on the lateral, medial, and dorsal surfaces of the left and right hemispheres. (Figure adapted from Vincent et al., 2008).

Resting-state functional connectivity analysis (rs-fcMRI) has proven to be particularly efficient in identifying large-scale polysynaptic cortical circuits, some of which have been defined in earlier work. According to Fox et al. [30] and Vincent et al. [29], the three major circuits are the dorsal attention system (DAS), the hippocampal-cortical memory system (HCMS), and the fronto-parietal control system (FPCS) (see Figure 4, adapted from Vincent et al., 2008, [7]).

Corbetta and Shulman [28] functionally described the DAS and the HCMS, based on earlier studies using classical fMRI paradigms. The DAS, consisting of portions of the intraparietal and superior frontal cortices, is involved in goal-directed top-down selection, whereas the HCMS, a ventral fronto-parietal network, subserves the bottom-up detection of salient or unexpected stimuli. Further, the HCMS has a role as an alerting system for the DAS in cases where the perceived signals are outside the current focus of attention [28]. An extension of the attention and perception framework [28] to episodic memory was proposed by Cabeza et al. [31], by re-evaluating the role of the parietal cortex. According to Cabeza and colleagues, the superior parietal cortex (SPL), as part of the DAS, should subserve top-down memory retrieval, search and verification, and the inferior parietal cortex (IPL), as part of the HCMS, should be engaged in high-confidence recollection.

The interaction between attention and memory, as a possible fundamental mechanism underlying working memory (WM), is increasingly being investigated. Bledowski et al. [32] hypothesized two complementary operations in WM: one for retrieval and one for updating of the attentional focus. Their fMRI results showed transient activations in the caudal superior frontal sulcus (cSFS) and the posterior parietal cortex (PPC) for updating, and, in contrast, a modulation specifically involving the rostral superior frontal sulcus (rSFS) and posterior cingulate/precuneus for selection. The combined results of a number of studies [28], [29], [31], [32] lead to the conclusion that WM emerges from the interaction of neuroanatomically dissociable components that are part of superordinate structures such as the DAS and the HCMS.

The third network found, the frontoparietal control system (FPCS) [29], is neuroanatomically located between the DAS and the HCMS, and it includes the lateral prefrontal cortex, the anterior cingulate cortex and the inferior parietal lobule. These regions have previously been linked with cognitive control and decision-making [4], [8], [9], [11], [33], [34]. The functional role of the FPCS is assumed to integrate information from the anti-correlated dorsal-attention and hippocampal-cortical memory systems [29], [30].

Remarkably, the perisylvian language network, consisting of the IFG, i.e., Brocas area (BA 44/45) and the superior temporal gyrus (STG) of Wernickes area (BA 42/22), is not covered by the three networks discussed. Rather, this network has been identified as the default language network based on low frequency fluctuation data across different language experiments by Lohmann et al. [35].

### Differences in the early short vs. the long period

After having established the base network with its three systems as a theoretical perspective, we are now in a position to apply it to the discussion of our experiment. Because all our formulae were visually presented, subjects had to first perceive and select the relevant information, and then mentally transform it, before a final stage of processing of the actual content could take place (cf. [4]). Therefore, we argue that the observed differences between the true algebraic and arithmetic stimuli for the early phase (4 s), reflect differential resource demands for bottom-up processes subserved by the ventral fronto-parietal network, the HCMS, for algebraic symbols compared to numbers. First, this interpretation is supported by the explicit presence of bilateral activation in the parahippocampal gyri (PhG), which served as the seed regions to identify the HCMS [29], [31] (cf. Table 1). Second, as put forward by Aminoff et al. [36], the PhG plays a central role in contextual associative operations, in addition to processing episodic memories or place related information. Further, the parietal activations we found were located in the ventral parietal cortex, namely in portions of the precuneus and the right inferior parietal lobule (IPL)/the superior temporal gyrus (STG), also called the temporoparietal junction (TPJ) area. Remarkably, as predicted by the neuroanatomical model of attentional control [28], [31], these parietal activations were indeed largely lateralized to the right (cf. Table 1). Also, the bilateral activation foci in the rostral portion of the superior frontal/middle frontal gyrus (BA 8/6) were located at the intersection of the HCMS and the FPCS [25], [29], an area which in the WM framework is thought to subserve selection processes [32]. Finally, activations in the fusiform gyrus, the visual module [4], reflected the reading of the stimuli items. All these regions are activated more for formulae containing algebraic symbols than for those containing numbers.

The same contrast evaluated for the 8 s after stimulus onset, showed a shift from inferior to superior regions in the parietal cortex and substantial additional differences for algebraic vs. arithmetic tasks in the left and right lateral prefrontal cortex (LPC) and the medial cortex (MFC) (see Table 2). This displacement can be interpreted as an increased reliance on top-down and control processes because the observed foci were situated within the frontoparietal control system (FPCS) and regions intersecting the dorsal attention system (cf. Table 2 and [29]). Again, in terms of WM, this would correspond to areas associated with the updating of information in contrast to the previous substrates involved in selection [32]. The transition from bottom-up to top-down processes is also clearly observable in the fusiform/middle occipital gyrus (BA 37/19). Whereas in the early phases the peak activation in BA 37 was more anterior (y = −42), it later shifted to more posterior regions (y = −57), and instead of the bilateral PhG activations which vanished, we found the middle temporal complex (MT+; y = −68) to be involved (cf. Table 2).

Taken together, these findings support the claim that the processing of the mathematical formulae reflects the temporal dependence on different cortical systems, initially operating bottom-up and later top-down.

### The prefrontal cortex and the rostro-caudal gradient

The prefrontal cortex (PFC) is vital for organizing human behavior and higher cognitive functions because it hosts some of the major components of the subserving faculties, such as working memory and cognitive control [4], [8], [9], [10], [25], [27], [29], [32], [37]. As expected, when compared to baseline all mathematical conditions yielded extended activation clusters in the medial, and in the lateral ventral and dorsal prefrontal cortices (cf. Figure 1).

In the medial prefrontal cortex (MFC) the active area observed was situated in the rostral cingulate zone (RCZ) [11], a connected region that intersects the pre-SMA (BA 6) and the mid-MFC (dACC; BA 32). Functionally, the RCZ is known to subserve performance monitoring, regulating cognitive control, and motivating ongoing processes [4], [9], [10], [11], [38]. The additional comparison of the true algebraic vs. the true arithmetic formulae, evaluated for the 8 s after stimulus onset, revealed that within the RCZ the algebraic condition involved an additional portion of the dACC. The arithmetic formulae did not require this extra volume as activation in this region was not present in the comparison of arithmetic true vs. baseline. The respective cluster boundaries in the anterior-ventral direction within the dACC were in Talairach space at 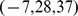 for algebra and at 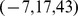 for the arithmetic stimuli; both vs. baseline.

This finding is meaningful because it provides an independent test for some theories concerning the organization and interaction of cognitive control and motivation in the PFC. Kounheiher et al. [10] showed that the medial and lateral PFC are functionally dissociable and that both follow a parallel organization, which is hierarchical along a posterior to anterior axis (cf. also [8], [25], [39]). This architecture implements two levels of processing hierarchies. At a first level, the pre-SMA transiently regulates, in response to contextual information or motivational incentives, the bilateral posterior lateral LPFC (LPFC), which is known to subserve contextual control. At a next level, the mid-MFC (dACC) drives in a sustained fashion, as a function of episodic information or motivational cues, the bilateral mid-LPC (BA 46/9), which is known to underlie episodic control [9], [10].

Within the first 4 s after stimulus onset no activation differences in the MFC for the true algebraic minus the true arithmetic stimuli would imply that no differences in the bilateral LPFC should be observable, which was in fact the case (cf. Table 1). On the other hand, for the same contrast taking the 8 s after stimulus onset into account, the measured difference in the dACC would imply that the mid-LPFC involvement should also be observable. Indeed, this was the case, as we found significantly increased activations for true algebraic vs. true arithmetic expressions in left and right BA 46/9 (cf. Table 2). Thus, the calculation of the truth value for both mathematical formulae types required MFC substrates, however, the “more abstract” (algebraic) expressions needed added and increased sustained episodic control, i.e., dACC involvement, in order to drive operational activities in the mid-LPFC. This could explain why the more abstract formulae are perceived as more difficult.

The pattern of activation foci in the LPFC (cf. Table 2), strikingly followed the dorsal “rostro-caudal functional gradient” [8], [9], [25], [39], which is known to be hosted by the frontoparietal control system (FPCS) (cf. [8]). Anatomically, the full axis proceeds from the dorsal premotor cortex (PMd) (BA 6) to the prePMd/post-LPC (BA 8/9/44), then to the mid-dorsolateral PFC (BA 46/9), and finally to the frontopolar cortex (FPC) (BA 10). As argued by several authors [8], [9], [25], [39], [40], the anatomy should mirror the functional hierarchies in various cognitive domains such as, e.g., WM [40], relational complexity [39], levels of abstraction [25], or prefrontal executive function [9].

The question that arises is how the processing of mathematical formulae fits into the rostro-caudal gradient framework. We propose an extension of Koechlin and colleagues' [9] cascade model from cognitive control to general information processing, which yields a good account with a strong theoretical underpinning. The original model [9] contains three nested levels for contextual, episodic and branching control, and an additional one for sensorimotor control, which are all supported by neural substrates along the dorsal axis, from the posterior to polar LPFC. In detail, sensory control is executed in the PMd (BA 6), and contextual control is hosted by the prePMd/post-PFC (BA 8/9/44), and episodic control is hosted by the dorsolateral PFC (BA 46/9), and branching control is hosted by the polar PFC (BA 10).

In our study we did not observe activations in the polar PFC, not even against baseline. We, therefore, conclude that this region does not seem to be required to process our hierarchical mathematical formulae, at least when the processing is straightforward (see Behavioral results). Thus, the role of the polar PFC indeed seems to be restricted to control purposes, such as conditional branching, cf. [9], [34].

However, all mathematical tasks showed, compared to baseline, significant involvement of the DLPFC, and the rostral part of the dorsolateral premotor cortex PMd (BA 6). This suggests an involvement of three regions for the processing of hierarchically structured formulae, as predicted for sequences requiring contextual and episodic control [8], [9]. These areas were activated more for algebra true vs. arithmetic true during the 8 s after stimulus onset, indicating an involvement of these regions in late top-down processes. Activations in the PFC have been reported in a number of different studies with respect to working memory, cognitive control and rule-based processes. Activations in the mid-LPFC (BA 46/9) have been linked to memory encoding and retrieval processes, in particular when organization, verification, and maintenance of structured information are required [27], [41], [42], [43], but also with dimensional [25] or episodic manipulations [9].

The bilateral anterior prePMd/post-LPFC (BA 8/9/44), as previously reported for contextual control [8], [9], [10], [25], partly overlaps with the inferior frontal junction area (IFJ), located at the intersection of the inferior frontal and inferior precentral sulci. The IFJ has been found to play an important role in task switching, set shifting and cognitive control, e.g., the Stroop task (cf. the review by Brass et al. [33]). Using a mental arithmetic task, Montojo and Courtney [44] showed, that updating of sequentially presented numbers (stimuli) and arithmetic operations (rules) both activated a common set of frontal and parietal regions, but that within this cortical network, number updates showed stronger activity in parietal regions (IPS) and rule updates in the vicinity of the left IFJ. Utilizing numerical, verbal, and spatial tasks, Hanakawa et al. [45] found that the PMd in conjunction with the PPC was also active outside the classical motor domain, and therefore, concluded that this region has an important cognitive function by associating rule-based symbolic cues across domains. These findings agree with the more recent ones indicating that these two regions are involved in updating information in WM [32].

The functional mapping we propose for the processing of mathematical formulae is as follows: First, it associates sensorimotor control areas with general information update processes (e.g., stimuli) in accordance with models from the theory of WM [32], [45]. Next, (mathematical) rules would correspond to contextual information, in line with previous research [10], [26]. Then, episodic information would correspond to data that might actually be transformed or generated, such as in logical or arithmetic operations within short-term memory [10], [37], [42]. Finally, the polar LPFC would remain a pure control area [9], [34].

The most rostro-ventral activation cluster we measured for the truth value of formulae was located in the left anterior ventrolateral prefrontal cortex (VLPFC; BA 47). Activation in this region was found in the comparison of true algebra vs. true arithmetic but also for the comparison of false vs. true arithmetic. Activations in the left anterior inferior gyrus (IFG), (BA 47/45) have been reported for the processing of semantic decision problems in the language domain [for a review see Poldrack et al. [46]. Outside the language domain BA 47 has been reported to support processing of mathematical logic [7] and problem solving in general [19]. The present results suggest that left BA 47 is involved in processing the semantic content of mathematical formulae.

BA 47 appears to be recruited when processing novel complex or abstract sequences. Note that the VLPFC (BA 47) is not considered part of the dorsal gradient, but rather part of a ventral system [8], [9], [25], with BA 47 acting as a memory controller during access and retrieval of knowledge stored in left temporal cortex (BA 21/22) (cf. [41], [47]).

Although the relation between the dorsal and the ventral gradient are not yet fully understood [25], the VLPFC seems to be invoked during complex tasks [7], [41], [46], [47] to support ongoing prefrontal processes by controlling and structuring strategic memory search.

### Parietal activations and the number sense

The IPS has been claimed to host the module for the number sense [1], [2], [5]. However, in the present study the algebraic condition, which involved no numbers, showed significant activations compared to both the baseline and the arithmetic conditions in the IPS. In our previous study on the syntax of flat and hierarchical formulae, the same observation was made, although none of the stimuli items involved numbers [7]. This suggests that the role of the IPS cannot be number-specific, but has to be considered to be more general (cf. also [6]).

Activations in the parietal cortex tend to co-activate with prefrontal regions, as has been observed in numerous studies investigating the vital functions subserving higher cognition, such as attention, organization, updating, selection, search, memory retrieval etc. [4], [6], [26], [28], [29], [30], [31], [43], [44], [45], [48]. Therefore, it is assumed that the parietal cortex has a role in subserving processes in prefrontal regions, by structuring and maintaining information that can be passed on [26], [43], [48].

In particular, the IPS has been reported by Cusack et al. [49] to be involved in structuring information into discrete and independent entities, so-called objects, which then can be manipulated separately. This data processing faculty is a prerequisite for many basic cognitive functions, e.g., attention or working memory. Therefore, in order to reconcile the diverging and contradictory findings concerning the strict number sense and the IPS, we propose the consideration of (small) numbers as elementary objects, which one might call “number objects”, and which represent a very simple form of structured information, made available for prefrontal regions.

## Conclusions

We have argued that the processing of mathematical formulae in the human brain can be decomposed into basic operations, such as working memory or cognitive control, which underlie this higher cognitive function. Temporally, different mechanisms for early bottom-up vs. late top-down processing can be observed, indicating shifts of the processing load within the base cortical network with pre-specified functional roles. Although the picture of a common macroscopic fronto-parietal network arises for the processing of the truth value of structurally identical mathematical formulae, the domain over which they are interpreted differentially modulates certain regions within this neural network. This is particularly pronounced in prefrontal regions, where the more abstract formulae require more (control) resources, which might indicate why the more abstract formulae are perceived as more difficult. Generally, this might explain why concrete examples can help when it comes to processing mathematical formulae.

## Materials and Methods

### Subjects

#### Fifteen

participants from the MPI CBS regular Human Subject Pool took part in this experiment (mean  = 25; 7 years old, SD  = 2.1, age range  = 23–30 years old, 4 female). All subjects were right handed and native German speakers with normal or corrected to normal vision. Each participant gave his/her informed consent, after having red and signed the Max Planck Institute for Human Cognitive and Brain Sciences' guidelines for fMRI studies. The experimental procedures were approved by the local Ethics Committee of the University of Leipzig.

### Stimuli

Our expressions were either true or false abstract algebraic or arithmetic formulae, e.g.,
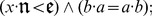
or the structurally equivalent arithmetic expressions, e.g.,



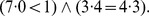



The stimuli were matched in terms of structure, number of characters and semantic content, i.e., Boolean truth value.

Our formulae were based on an alphabet consisting of variables: 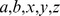; numeric constants: 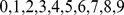; algebraic constants: additive neutral element 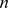 and the unit for multiplication 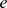; logical symbols (and, or): 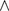, 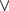 equality 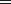; left 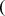, right 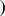 parenthesis; binary relation symbol: 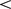 (“less than”); two binary function symbols (plus, multiplication): 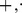 . The set of variables and constants was chosen randomly, whereas the selection of the other symbols followed specific rules. Out of the symbols, we built first order formulae, each of them involving exactly 17 symbols, that represented unambiguously either true or false statements. In total, there were 25 true algebraic versus 25 false algebraic formulae and equivalently 25 true arithmetic versus 25 false arithmetic formulae. All stimuli could be represented by binary trees of the four following types (1, 11, 0111; 1, 11, 1011; 1, 11, 1101; 1, 11, 1110). The task was to decide whether the expression shown represented a true or false statement. For the baseline image, a row of white-grayish circles, on a very dark gray background was used.

### fMRI Acquisition

The software packages used were LIPSIA [50] for the data analysis and PRESENTATION (Neurobehavioral Systems) for the visual presentation of the stimulus material. The study was conducted on a 3T BRUKER scanner (Medspec S300, Bruker, Ettlingen).

For registration purposes, two sets of two-dimensional anatomical images were acquired for each participant immediately prior to the functional imaging. An MDEFT and an EPI-T1 sequence were used. T1-weighted MDEFT images were obtained, with a non slice-selective inversion pulse followed by a single excitation of each slice. Anatomical images were positioned parallel to AC-PC. The functional MRI parameters were as follows; Axial slices: TR  = 2 s, TE  = 30 ms, alpha  = 90°, 29 slices (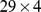 mm  = 11.6 cm, whole brain), 4 mm slice thickness (no gap), voxel volume: 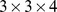 mm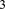, 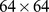 matrix, 19.2 cm FOV. There were 25 stimuli per condition (4 conditions +nullevent), presented with SOA  = 11 s, with a total stimulation time of 27 minutes (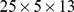 seconds).

### fMRI Analysis

The data processing was performed using the software package LIPSIA [50]. This software package contains tools for preprocessing, co-registration, statistical evaluation, and visualization of fMRI data. Preprocessing was carried out as follows: Functional data were motion-corrected using a matching metric based on linear correlation. To correct for the temporal offset between the slices acquired in one scan, a cubic spline-interpolation was applied. A temporal high-pass filter with a cut-off frequency of 1/72 Hz was used for baseline correction of the signal and a spatial Gaussian filter with 6 mm FWHM was applied. The increased auto-correlation caused by the filtering was taken into account during statistical calculation by an adjustment of the degrees of freedom.

Subsequently, co-registration of data was carried out. To align the functional slices with a 3D stereotactic coordinate reference system, a rigid linear registration with six degrees of freedom (3 rotational, 3 translational) was performed. The rotational and translational parameters were acquired based on the MDEFT and EPI-T1 slices to achieve an optimal match between these slices and the individual 3D reference data set. This 3D reference data set was acquired for each subject during a previous scanning session. The MDEFT volume data set with 160 slices and 1 mm slice thickness was standardized to the Talairach stereotactic space [20]. The rotational and translational parameters were subsequently transformed by linear scaling to a standard size. The resulting parameters were then used to transform the functional slices using trilinear interpolation so that the resulting functional slices were aligned with the stereotactic coordinate system. This linear normalization process was improved by a subsequent processing step that performed an additional non-linear normalization.

The statistical evaluation was based on a least-squares estimation using the general linear model for serially auto-correlated observations. The design matrix was generated with a synthetic hemodynamic response function and its first and second derivative. The model equation, including the observation data, the design matrix and the error term, was convoluted with a Gaussian kernel of dispersion of 4 s FWHM to deal with the temporal auto-correlation. Afterwards, contrast-images (i.e., estimates of the raw-score differences between the specified conditions) were calculated for each subject. Each individual functional data set was aligned with the standard stereotactic reference space, so that a group analysis based on the contrast-images could be performed.

The individual contrast-images were first masked and the individual and masked contrast-images were then entered into a second-level random effects analysis (one-sample 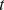-test). Subsequently, 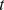-values were transformed into 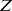-scores.

Then we performed a multiple comparison correction, based on Monte-Carlo simulation, which used a combination of individual voxel probability thresholding and minimum cluster-size thresholding. The uncorrected probability threshold was set to 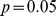. For the determination of the minimal cluster-size we referred also to a Monte-Carlo simulation, with the cluster size being associated to the largest corrected 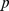-value 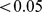, which resulted in regions with at least 297 mm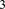 to be considered. The group analysis was performed by averaging individual 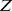-maps and multiplying each 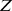-value with the square root of the number of subjects in the experiment.

### Procedure

The experiment was devised as a reading experiment with button press used to indicate the result of the evaluation (true or false).

The 100 experimental hierarchical stimuli items (50 stimuli of algebraic type, with no numbers and 50 stimuli of arithmetic type, with numbers but with no algebraic variables or constants) and 25 identical baseline stimuli (a row of white-grayish circles on a very dark gray background) were presented in a fully randomized order, for a fixed period.

Algebraic stimuli were visible for 12 s, arithmetic stimuli were visible for 9 s, and baseline events were visible for 9 s. The subjects' task was to judge the Boolean truth value, i.e., true vs. false, of every formula shown.

A response had to be given for every stimulus of a formula type, after the stimulus item disappeared from the screen and a new screen indicated that the answer had to be given. The participant had 2200 ms to press the respective button, i.e., one for “true” and one for “false”. No feedback was given after the button-press. For the baseline condition, no answer was required.

All the material to be presented, including the visibility of the stimuli, was piloted in the scanner. All participants were carefully instructed before the actual test, and had a training session with a sample of similar stimuli presented on a laptop where they responded with a button press device.
