# Correction: Mathematical Logic in the Human Brain: Semantics

**DOI:** 10.1371/annotation/c08ee740-5917-4096-97e9-378ae5be1208

**Published:** 2013-08-21

**Authors:** Roland M. Friedrich, Angela D. Friederici

There were multiple errors in the affiliations. The correct affiliations are:

Roland M. Friedrich

Institute for Mathematics, Humboldt University Berlin, Berlin, Germany and

Department of Neuropsychology, Max Planck Institute for Human Cognitive and Brain Sciences, Leipzig, Germany

Angela D. Friederici

Department of Neuropsychology, Max Planck Institute for Human Cognitive and Brain Sciences, Leipzig, Germany 

